# *In vitro* reconstitution of the bacterial cytoskeleton: expected and unexpected new insights

**DOI:** 10.1111/1751-7915.13336

**Published:** 2018-11-08

**Authors:** Beatrice Ramm, Petra Schwille

**Affiliations:** ^1^ Max Planck Institute of Biochemistry Am Klopferspitz 18 D‐82152 Martinsried Germany

## Abstract

*In vitro* reconstitution of bacterial cytoskeletal elements, primarily supposed to reveal detailed mechanistic insights, has been an invaluable source of unexpected new protein functionalities. This may be particularly beneficial in the context of a potential construction of artificial cells from the bottom‐up.

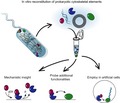

*In vitro* reconstitution of bacterial cytoskeletal elements, primarily supposed to reveal detailed mechanistic insights, has been an invaluable source of unexpected new protein functionalities. This may be particularly beneficial in the context of a potential construction of artificial cells from the bottom‐up.

Every living cell needs to organize its content in space and time. To achieve this, cells have developed sophisticated nanomachineries consisting of cytoskeletal filaments, motor proteins, as well as reaction‐diffusion type systems of soluble molecules. Research into these systems has a long history in eukaryotic cells. Next to studying the dynamic processes in living cells, classic biochemical reconstitution has emerged early on as a valuable tool to elucidate mechanistic details of individual protein systems, such as actin and myosin (Kron and Spudich, 1986; Liu and Fletcher, 2009). Recently, the field of eukaryotic reconstitution has moved towards constructing ever more complex phenomena with increasing numbers of molecular components *in vitro* such as the reconstitution of T cell receptor signalling (Su *et al*., 2016). This increase in complexity culminates in the vision of ultimately creating a living cell from the bottom‐up (Schwille, 2015).

In contrast, prokaryotes have long been ridiculed as simple bags of molecules with a lack of intracellular organization. Only the recent advances in high resolution microscopy techniques have changed that perception and revealed that also bacteria have intricate mechanisms and structures to organize their cellular components. Hence, biochemical reconstitution of prokaryotic cytoskeletal elements started with much delay around 10 years ago. Since this field is thus still in its infancy, we expect a burst of new structural and mechanistic insights into the inner workings of bacteria in the coming years (Fig.1).

**Figure 1:**
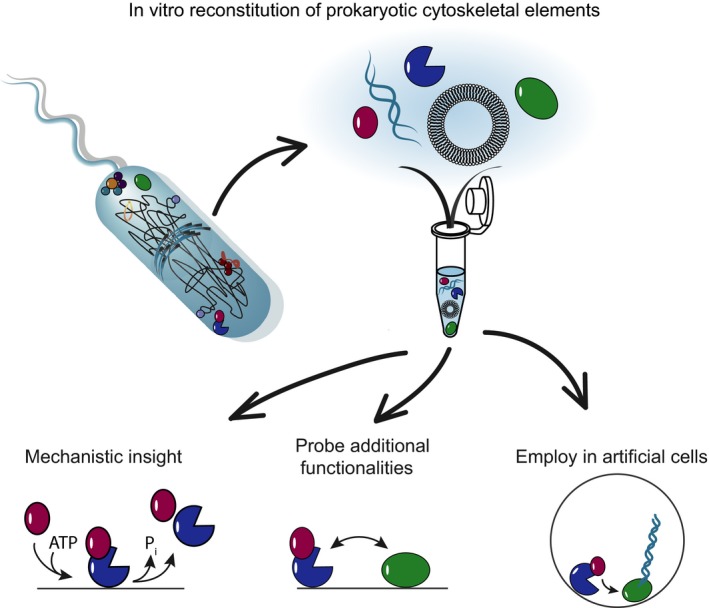
*In vitro* reconstitution of prokaryotic cytoskeletal elements will generate mechanistic insight, allow to probe additional functionalities, test hypothesis and will be employed in artificial cells in a potentially new context/function.

In particular, future studies using biochemical *in vitro* reconstitution of prokaryotic modules will shed light onto the detailed active mechanisms of cytoskeletal organization and transformation, and of reaction‐diffusion systems, highlighting similarities and differences to their eukaryotic counterparts. Past examples of this approach already provided insight into DNA segregation by the actin homologue ParM (Garner *et al*., 2007), the treadmilling dynamics of the tubulin homologue FtsZ (Loose and Mitchison, 2014; Ramirez‐Diaz *et al*., 2018) or the minus end‐tracking system TubZRC (Fink and Löwe, 2015).

Notably, once an *in vitro* assay has been established for a particular protein system, it can be used to screen for additional hidden functions or interaction partners of the proteins, or to test hypotheses regarding their roles in the cell. A prime example for this line of research comes from the *in vitro* reconstitution of the *E. coli* MinCDE system. In the cell, MinD and MinE oscillate from pole to pole ‘piggybacking’ a third protein MinC which establishes a time‐averaged protein gradient of MinC that inhibits FtsZ polymerization. Several years ago, this protein self‐organization was reconstituted *in vitro,* where MinD and MinE form fascinating travelling surface waves and pole‐to‐pole oscillations on model membranes (Loose *et al*., 2008). The assay has since served to elucidate the detailed mechanism of pattern formation by MinDE (Loose *et al*., 2011; Vecchiarelli *et al*., 2014). However, it has always been astonishing that the cell would entertain energy‐consuming large‐scale oscillations in order to position its division ring. Indeed, *in vivo* studies provided several hints for the involvement of Min oscillations in other processes than FtsZ regulation, such as the targeting of peripheral membrane proteins, or chromosome segregation. However, clear evidence could not be gained from cellular studies, owing to their compositional complexity. Taking advantage of the reduced complexity *in vitro*, the established assay was recently employed to show that MinDE oscillations can serve to position functionally unrelated membrane‐bound proteins into patterns and large‐scale gradients by forming a propagating diffusion barrier (Ramm *et al*., 2018). This finding was highly unexpected, and the observed non‐specific mechanism of active protein transport by MinDE has so far no known counterpart in eukaryotic cell biology. It impressively demonstrates that *in vitro* assays not only have the power to quantitatively characterize known mechanisms relevant for prokaryotic cell biology but, due to the greater simplicity of the systems, will uncover fully new mechanistic concepts that might even be conserved in eukaryotes, but are currently hidden behind their compositional complexity.

Finally, bacterial cytoskeletal components may be engineered and employed in a new context towards the bottom‐up construction of artificial cells. One example is the recent development of a photoswitchable MinDE system to control pattern formation (Glock *et al*., 2018). Additionally, how the self‐organizing properties of the bacterial cytoskeleton can be diverted towards new use was lately demonstrated using the polymeric features of FtsZ. In *E. coli,* FtsZ interacts with the membrane via its anchors FtsA and ZipA, and recruits other divisome components into a filamentous ring structure to eventually constrict the septum. However, when the protein was reconstituted in membrane‐less coacervate droplets (Te Brinke *et al*., 2018), FtsZ polymerization deformed these coacervate compartments and, when GTP supply was anisotropic, even formed elongated fibrils that led to division events of the coacervate‐FtsZ phases.

While the quest for artificial cells avails itself of eukaryotic, prokaryotic and purely synthetic systems, we believe that the prokaryotic cytoskeleton will continue to prove a true treasure chest for this endeavour. The already large variety of different cytoskeletal elements and nucleotide‐dependent switches is very likely to be expanded by the discovery of new types from the countless bacterial species (Surovtsev and Jacobs‐Wagner, 2018; Wagstaff and Löwe, 2018). Homologous systems from different species might supply a plethora of non‐cross reacting modules that can be combined to achieve similar tasks, for example segregation of chromosomes or other functional macromolecules within the same artificial cell. In contrast to this system diversity stands the relative compositional simplicity of prokaryotic systems compared to eukaryotes, where usual 2–3 protein components work together to achieve nevertheless sophisticated tasks.

The full potential of prokaryotic reconstitution biology will be unfolded by harnessing cell‐free protein expression and microfluidics, providing the technical means to increase compositional complexity, but still retain precise control over the various components. With these tools in hand, reconstitution efforts should eventually lead to the bottom‐up construction of cell‐like entities. These have in the past been promoted in two ways: to help further deciphering fundamental physicochemical principles of life, but also as simple and potentially more efficient biotechnological reactors without the drawbacks of evolved systems: pathway redundancy and inefficient energy consumption. Furthermore, bottom‐up assembled artificial cells based on prokaryotic elements might serve as a testbed for industrial and academic research, as all components are well‐defined and quantitatively understood. For example, it could tremendously aid metabolic pathway modelling and help to identify bottlenecks in recombinant protein production. Last but not least, a prokaryotic synthetic cell could help to identify targets for novel antibiotics and test their potency.

To conclude, in the coming years, we will see a surge in reconstitution of bacterial protein functions that will not only give us (i) detailed mechanistic insight on their inner workings, but will also allow us to (ii) probe unexpected functionalities that are hard to decipher *in vivo* and (iii) employ proteins in a different context for applications in the bottom‐up construction of artificial cells.
